# Combined Exendin-4 and Rituximab Treatment Improves β-Cell Function and Delays Disease Progression in NOD Mice

**DOI:** 10.3390/biology15171472

**Published:** 2026-08-31

**Authors:** Yongbin Wang, Shan He, Huan Yang, Jie Zhang, Likun Yu, Jianjun Xiong, Xingnuan Li, Jiashuo Qiu, Baicheng Ma

**Affiliations:** 1 School of Health Science and Engineering, University of Shanghai for Science and Technology, South Campus of Jun Gong Road, Yangpu District, Shanghai 200093, China; wyb030115@163.com; 2 Jiangxi Provincial Key Laboratory of Cell Precision Therapy, School of Basic Medical Sciences, Jiujiang University, Jiujiang 332005, China; heshan320128@163.com (S.H.); yanghuan8818@163.com (H.Y.); bailihongchen@163.com (J.Z.); wuyuanylk@163.com (L.Y.); xiongjj1975@163.com (J.X.); 6140074@jju.edu.cn (X.L.); qiu3392906017@163.com (J.Q.)

**Keywords:** type 1 diabetes mellitus, rituximab, exendin-4, non-obese diabetic (NOD) mice, combined treatment

## Abstract

Type 1 diabetes mellitus is characterized by immune-mediated pancreatic β-cell loss and progressive impairment of insulin production. This study evaluated the effects of combined rituximab, a B-cell-directed therapy, and exendin-4, a glucagon-like peptide-1 receptor agonist, on β-cell-related function and metabolic outcomes in experimental type 1 diabetes. Pancreatic islets isolated from NOD-scid mice were used for in vitro assessment of viability, metabolic activity, insulin secretion under high-glucose conditions, and gene expression. Female NOD mice were assigned to control, exendin-4, rituximab, or combination treatment groups and treated for approximately 20 weeks. Combined treatment showed the greatest increases in insulin-related responses in vitro. In vivo, it was associated with more stable fasting blood glucose levels, improved oral glucose tolerance, delayed progression to hyperglycemia, increased circulating insulin and C-peptide levels, and reduced glucagon, HbA1c, and LDL levels. Histological and molecular analyses further demonstrated reduced pancreatic inflammatory damage, reduced apoptosis-associated changes, and increased BrdU- and cytokeratin-18-associated signals within insulin-positive islet regions. These findings support further investigation of combined B-cell-directed and β-cell-supportive strategies for preserving islet function and delaying disease progression in type 1 diabetes.

## Simple Summary

Type 1 diabetes develops when the immune system damages the insulin-producing cells of the pancreas, leading to a progressive loss of insulin production and poor blood sugar control. In this study, we investigated whether combining rituximab, an antibody-based treatment previously investigated in type 1 diabetes, with exendin-4, a drug that supports insulin-producing cell function, could provide greater benefits than either treatment alone. Experiments were performed using isolated pancreatic islets and a mouse model of type 1 diabetes. The combined treatment improved insulin-related responses, helped maintain more stable blood glucose levels, improved glucose tolerance, and delayed the development of high blood sugar. It was also associated with better preservation of pancreatic islet structure, reduced inflammatory damage, and fewer signs related to cell death. These findings support further investigation of combined rituximab and exendin-4 treatment as a potential strategy for preserving islet function and delaying disease progression in early-stage type 1 diabetes.

## 1. Introduction

Type 1 diabetes mellitus (T1DM) is an autoimmune disease caused by interactions between genetic susceptibility and environmental factors and is associated with an insulin deficit resulting from immune-mediated pancreatic β-cell loss [1,2]. From a clinical perspective, affected individuals typically present with hyperglycemia accompanied by markedly reduced or undetectable C-peptide levels [3]. The global incidence of T1DM continues to increase under the influence of multiple factors [4]. The pathogenesis of T1DM is mainly driven by immune imbalance, particularly excessive autoreactive CD8^+^ T-cell-mediated cytotoxicity together with impaired immune tolerance mechanisms [5]. In this process, B cells and dendritic cells (DCs) act as antigen-presenting cells that present β-cell autoantigens to CD4^+^ and CD8^+^ T cells, thereby promoting and maintaining islet inflammation and also contributing to the formation of islet-specific autoantibodies [6]. Together, these findings suggest that targeting B-cell-mediated immune injury has potential in treating the disease.

Despite the established role of exogenous insulin therapy as the standard of care for T1DM, this approach remains limited in its ability to address the underlying autoimmune pathology or halt progressive loss of residual β-cell function. Consequently, patients remain at risk of poor glycemic control and long-term complications [7]. In recent years, immunomodulatory therapies have gained increasing attention as potential disease-modifying approaches in T1DM. Among them, rituximab, an anti-CD20 monoclonal antibody, has shown potential by depleting B cells and thereby reducing autoimmune-mediated β-cell injury [8,9]. However, immunotherapy alone may be insufficient, particularly when β-cell mass is already significantly reduced. A major limitation of current immunomodulatory strategies is that, although they can attenuate autoimmune-mediated β-cell destruction, they have limited capacity to preserve residual β-cell function or compensate for established β-cell loss. Therefore, therapeutic approaches integrating immune modulation with interventions aimed at enhancing β-cell survival and function may provide a more effective strategy than immune-targeted therapies alone.

Agonists of the glucagon-like peptide-1 (GLP-1) receptor influence β-cell function beyond glucose lowering [10,11]. One such agonist, exendin-4, enhances insulin secretion, promotes β-cell survival, and contributes to the preservation of β-cell functional capacity [12]. Several studies have investigated combination strategies such as exendin-4 with anti-CD3 monoclonal antibody [13], and liraglutide with anti-IL-21 monoclonal antibody [14], which showed significant therapeutic effects, and other combinations have also shown promising results [15,16]. Therefore, combining immune modulation with β-cell protection may be more effective than monotherapy alone.

However, the therapeutic potential of combining GLP-1 receptor agonism with B-cell-directed immunomodulation remains insufficiently explored in T1DM. Unlike strategies targeting immune dysregulation or β-cell support individually, this combination approach aims to integrate a B-cell-directed therapeutic strategy with support for residual β-cell function. Therefore, we hypothesized that combined rituximab treatment and exendin-4-mediated GLP-1 receptor activation would produce greater therapeutic effects compared with either intervention alone. In this study, isolated-islet assays were used to assess islet-level responses, whereas NOD mice were used to evaluate effects on β-cell functional capacity, metabolic regulation, and disease progression in experimental T1DM.

## 2. Materials and Methods

### 2.1. Materials

A total of twenty-four eight-week-old female non-obese diabetic (NOD) mice and four 12-week-old NOD-scid mice were obtained from the Model Animal Research Center of Nanjing University. The NOD mice (*n* = 24) were acclimatized for one week before experimentation and housed in groups of six in each cage under a 12 h light/dark cycle with unrestricted water and standard chow.

Fetal bovine serum (16000-044, GIBCO, Grand Island, NY, USA), DMEM (SH30243.01, Hyclone, Logan, UT, USA), penicillin–streptomycin (100×) (P1400-100, Solarbio, Beijing, China), Ficoll^®^ 400 (F2637, Sigma, Virginia Beach, VA, USA), Collagenase P (11213857001, Sigma, Virginia Beach, VA, USA), 0.25% trypsin–EDTA (T1300-100, Solarbio, Beijing, China), MTT reagent (M5655, Sigma, Virginia Beach, VA, USA), DMSO (302A0316, Amresco, Solon, OH, USA), double sulfur brown (D9630, Solarbio, Beijing, China), Annexin V-FITC apoptosis assay kit (C1062M, Beyotime Biotechnology, Shanghai, China), Krebs–Ringer bicarbonate buffer (G0430, Solarbio, Beijing, China), Hank’s balanced salt solution (HBSS) (H6648, Sigma, Virginia Beach, VA, USA), exendin-4 (HY-13443, MCE, Monmouth Junction, NJ, USA), rituximab (100 mg/10 mL, Roche, Basel, Switzerland), mouse insulin ELISA test kit (ml001983-1, Shanghai Enzyme-Linked Biotechnology Co., Ltd., Shanghai, China), BrdU antibody (MA5-50785, Thermo Fisher Scientific, Waltham, MA, USA), insulin INS monoclonal antibody (66198-1-Ig, Proteintech, Rosemont, IL, USA), and cytokeratin antibody (10830-1-AP, Proteintech, Rosemont, IL, USA), CoraLite^®^ Plus 488 TUNEL apoptosis detection kit (PF00006, Proteintech, Rosemont, IL, USA), CoraLite^®^ Plus 594-goat anti-rabbit recombinant secondary antibody (RGAR004, Proteintech, Rosemont, IL, USA), and CoraLite^®^ Plus 488-goat anti-mouse recombinant secondary antibody (RGAM002, Proteintech, Rosemont, IL, USA) were used. Additional reagents and consumables were supplied by Wuhan BaiTai Science and Technology Co., Ltd., Wuhan, China. Statistical analyses and graphical presentations were performed using GraphPad Prism 10 (GraphPad Software, San Diego, CA, USA), while fluorescence image quantification was performed using Fiji (ImageJ 1.54r; National Institutes of Health, Bethesda, MD, USA).

### 2.2. In Vitro Experiments

Islets isolated from NOD-scid mice were used to establish a stable in vitro culture system for functional evaluation. Each in vitro assay was independently repeated three times using separately prepared islet cultures. For MTT, ELISA, and RT-qPCR analyses, each condition was run in triplicate technical wells. The mean of the technical replicates was used to represent one biological replicate, and statistical analyses were performed using three independent biological replicates (*n* = 3).

#### 2.2.1. Isolation and Culture of Islets

NOD-scid mice (*n* = 4) were anesthetized, and the abdominal and thoracic cavities were opened to enable cannulation of the common bile duct. A pre-cooled 1 mg/mL collagenase P solution was injected through the duct, and the pancreas was excised and incubated in pre-warmed Hank’s balanced salt solution for 10 min at 37 °C. The tissue was gently dissociated using forceps, and digestion was stopped by adding HBSS containing FBS. The suspended cells were filtered (600-µm mesh) before centrifugation (1000 rpm at 4 °C for 2 min).

The pellet was washed with Hank’s balanced salt solution, resuspended in 25% Ficoll, and sequentially layered with 23% to 20% and 20% to 11% Ficoll solutions, followed by Hank’s balanced salt solution. The suspension was centrifuged (3000 rpm, 20 min, 4 °C), and the islets were collected from the interfaces between 23% and 20% and 20% and 11%. Isolated islets were transferred into T25 culture flasks containing 5 mL high-glucose DMEM supplemented with 10% FBS and 1% penicillin–streptomycin. The cultures were maintained at 37 °C in a humidified incubator with 5% CO_2_, with replacement of the medium every three days, and islet morphology and growth were regularly monitored.

#### 2.2.2. Dithizone (DTZ) Staining

Cultured islet clusters were identified using DTZ staining. The DTZ stock solution was prepared by dissolving 10 mg of DTZ in 10 mL of DMSO. The stock solution was diluted 1:1000 in PBS and filtered (0.22 μm membrane) prior to use. This solution was added to cultured islet cells for 10 min at room temperature, after which cells were evaluated and imaged under light microscopy (100× magnification).

#### 2.2.3. Flow Cytometric Analysis of Viability and Apoptosis

Following trypsin-EDTA digestion and washing with PBS, islet cells (1 × 10^5^) in 195 μL of Annexin V-FITC binding buffer were mixed gently with 10 μL Annexin V-FITC and 5 μL PI, maintained initially for 15 min at room temperature with light protection, and then on ice. Unstained cells served as negative controls. Flow cytometric analysis was performed, and cells were categorized as viable, early apoptotic, late apoptotic, and necrotic.

#### 2.2.4. MTT Assays

Cultured islet cells were digested with trypsin–EDTA and resuspended to 2 × 10^4^/mL. Cells (100 µL/well) were then inoculated into a 96-well plate, with three replicate wells per condition. Wells containing culture medium alone served as blank controls. Cells were treated with exendin-4 (1, 10, and 100 nM), rituximab (1, 8, and 16 mg/L), or combinations of rituximab and exendin-4 (1 mg/L + 1 nM, 8 mg/L + 10 nM, and 16 mg/L + 100 nM) and incubated at 37 °C for 12, 24, and 48 h. Subsequently, 20 μL MTT solution (5 mg/mL) was introduced to each well for 4 h. After careful removal of the medium, DMSO (150 μL) was applied for formazan crystal dissolution. Following shaking for 10 min, absorbances were read at 490 nm in a microplate reader. Cellular metabolic activity was expressed as absorbance values plotted against incubation time.

#### 2.2.5. Static Insulin Secretion Assay Under High-Glucose Conditions and ELISA

Purified islets were cultured overnight in 6-well plates prior to treatment. Cells were then exposed to exendin-4 (100 nM), rituximab (8 mg/L), or a combination of rituximab (8 mg/L) and exendin-4 (100 nM), while an equivalent volume of normal saline served as the control group. After 24 h incubation, islets were washed twice with culture medium and subsequently incubated in Krebs–Ringer bicarbonate buffer with 17 mM glucose for 1 h to evaluate insulin secretion under high-glucose conditions. Insulin levels in both culture supernatants and cell lysates were evaluated using a mouse insulin ELISA kit (ml001983-1, Shanghai Enzyme-Linked Biotechnology Co., Ltd., Shanghai, China), as directed, with three replicate wells per condition. Specifically, 50 μL of standards or samples was placed in each well before the introduction of 50 μL of biotin-labeled antibody for 1 h at 37 °C. After three rinses, 80 μL of streptavidin–HRP was introduced for 30 min at 37 °C. After further rinsing, substrate solutions A and B (50 μL of each) were added for 10 min at 37 °C with light protection. Fifty microliters of stop solution was added to stop the reaction, and absorbance was recorded at 450 nm. A standard curve was used for determining insulin concentrations.

#### 2.2.6. Reverse Transcription and Quantitative PCR Analysis

Total RNA was extracted from islet cells using TRIzol reagent according to the manufacturer’s instructions and reverse-transcribed into complementary DNA (cDNA). cDNA synthesis was carried out on a GeneAmp^®^ PCR System 9700 (Applied Biosystems, Foster City, CA, USA) under the following conditions: 42 °C for 60 min for reverse transcription, 70 °C for 5 min for enzyme inactivation, and 15 °C for 2 min for cooling, followed by storage at 4 °C until further use.

Quantitative real-time PCR was performed using SYBR^TM^ Green Real-Time PCR Master Mix (QPK-201, TOYOBO, Osaka, Japan) on a 7500 Real-Time PCR System (Applied Biosystems, Foster City, CA, USA). Thermal cycling conditions consisted of an initial denaturation at 95 °C for 60 s, followed by 40 amplification cycles of denaturation at 95 °C for 15 s and annealing/extension at 60 °C for 34 s. The mRNA expression levels of *insulin*, *Bcl2*, *Bax*, and *Caspase-3* were quantified, with *β-actin* serving as the internal reference. Amplification specificity was assessed by melting-curve analysis. Amplification efficiencies were calculated from standard curves generated using serial dilutions of cDNA according to the equation E = [10^^(−1/slope)^ − 1] × 100%. The primer sequences, amplification efficiencies, and corresponding R^2^ values are provided in Table 1. Relative expression was determined using the 2^^−ΔΔCt^ method. RT-qPCR was performed to evaluate the effects of rituximab and exendin-4 treatment on insulin gene expression and apoptosis-related molecular changes in islet cells.

### 2.3. In Vivo Experiments

#### 2.3.1. Experimental Design

**Figure 1:**
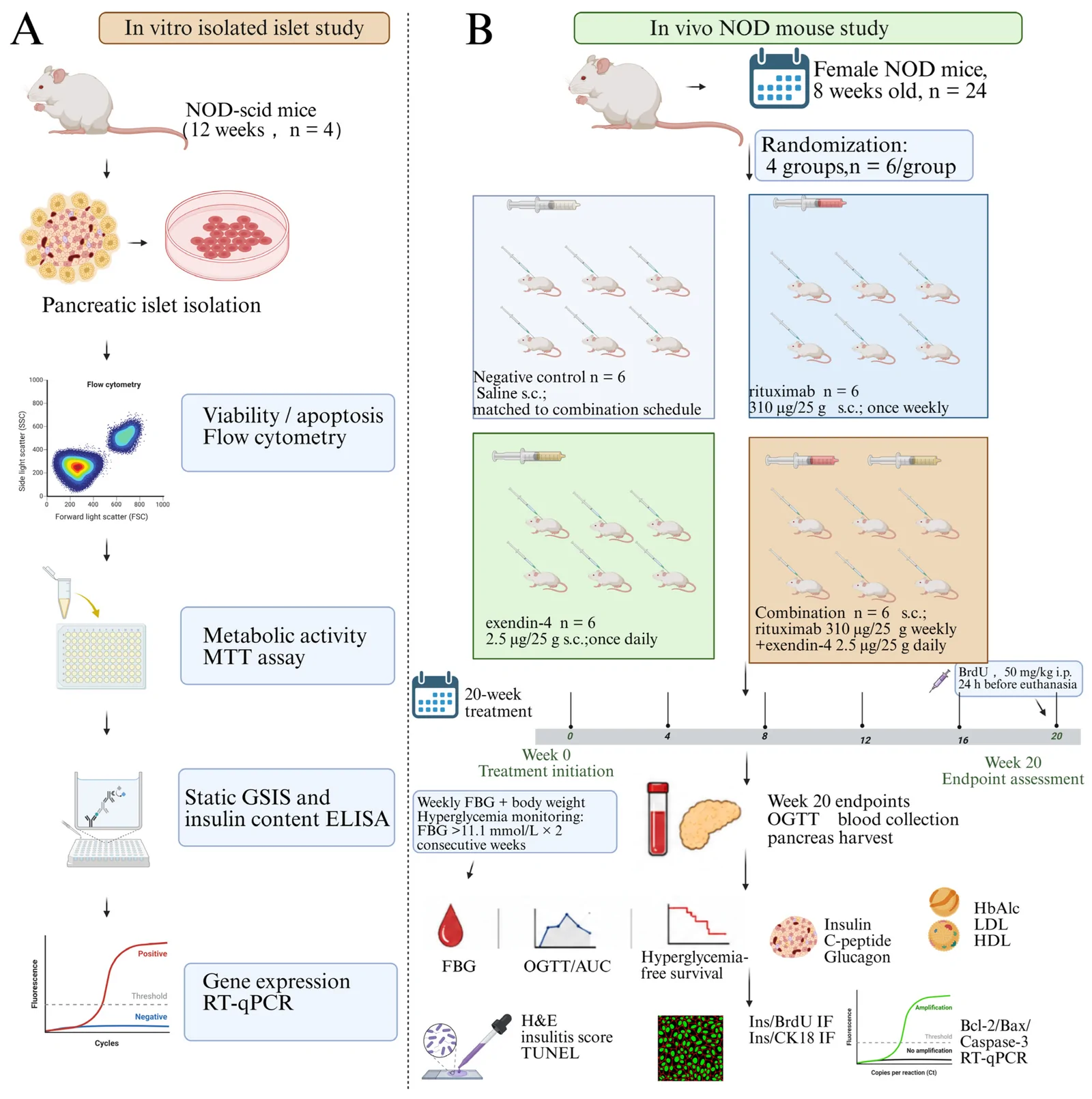
Schematic overview of the in vitro and in vivo study design. (**A**) In vitro isolated islet study. Pancreatic islets were isolated from 12-week-old NOD-scid mice and subjected to flow cytometric analysis of viability and apoptosis, MTT assay, static insulin secretion assay under high-glucose conditions (17 mM glucose), insulin content determination by ELISA, and RT-qPCR analysis of gene expression. (**B**) In vivo NOD mouse study. Eight-week-old female NOD mice were randomly assigned to four groups: control, exendin-4, rituximab, and combination treatment. The control group received saline according to the treatment schedule of the combination group. Exendin-4 was administered subcutaneously at 2.5 μg/25 g daily, whereas rituximab was administered subcutaneously at 310 μg/25 g weekly. Mice in the combination group received both treatments at the same doses and schedules. Treatment continued for 20 weeks. Fasting blood glucose levels and body weight were monitored weekly. Hyperglycemia was defined as fasting blood glucose ≥ 11.1 mmol/L in two consecutive weekly measurements. OGTT, blood collection, and pancreatic tissue collection were performed at the endpoint. BrdU was administered intraperitoneally (50 mg/kg) for 24 h before euthanasia. Endpoint analyses included glucose tolerance (OGTT/AUC), hyperglycemia-free survival, metabolic parameters, pancreatic histopathology, insulitis assessment, apoptosis analysis, immunofluorescence staining, and RT-qPCR analysis of apoptosis-related genes. Abbreviations: AUC, area under the curve; BrdU, 5-bromo-2′-deoxyuridine; CK18, cytokeratin-18; FBG, fasting blood glucose; HbA1c, glycated hemoglobin; H&E, hematoxylin and eosin; HDL, high-density lipoprotein; i.p., intraperitoneal injection; LDL, low-density lipoprotein; OGTT, oral glucose tolerance test; RT-qPCR, reverse transcription-quantitative polymerase chain reaction; s.c., subcutaneous injection.

All animal experiments were conducted and reported in accordance with the ARRIVE (Animal Research: Reporting of In Vivo Experiments) guidelines and the Guide for the Care and Use of Laboratory Animals, as well as relevant institutional regulations on animal welfare and ethics. The experimental protocol was reviewed and approved by the Laboratory Animal Welfare and Ethics Committee of Jiujiang University on 24 March 2023 (Approval No. 20230324). Female NOD mice were used in this study because they spontaneously develop autoimmune diabetes, and females show a higher disease incidence than males, making them a well-established model for T1DM research [17]. A total of 24 female NOD mice were included in the in vivo study, with six animals assigned to each experimental group. The group size was selected with reference to previous NOD mouse intervention studies assessing metabolic and histological outcomes, while also considering the principle of reducing animal use. No formal a priori power calculation was performed. After one week of acclimatization under a 12 h light/dark cycle with unrestricted water and food, mice were assigned unique identification numbers before treatment initiation. A random allocation sequence was generated using a random number table to assign mice to four treatment groups at a 1:1:1:1 ratio: control, rituximab, exendin-4, and rituximab plus exendin-4. Group allocation was completed before the first administration, and all subsequent treatments and longitudinal assessments were performed according to the assigned allocation. Fasting blood glucose levels were recorded before treatment initiation. Animals in the control group received saline. Exendin-4 was administered by subcutaneous injection at 2.5 μg/25 g once daily [18,19], and rituximab was administered by subcutaneous injection at 310 μg/25 g once weekly [20]. Mice in the combination treatment group received both agents at the same doses and schedules. The exendin-4 dose (2.5 μg/25 g, daily) was selected based on previously reported murine studies demonstrating its ability to activate GLP-1 receptor signaling and influence β-cell function. Rituximab was administered at 310 μg/25 g once weekly based on a previously described dosing regimen in NOD mice. The selected dosing regimens were applied throughout the experimental period to evaluate the combined effects of exendin-4 and rituximab treatment in experimental T1DM. Treatment continued for approximately 20 weeks. Fasting blood glucose levels and body weights were assessed each week, and an oral glucose tolerance test (OGTT) was performed before euthanasia. The overall experimental workflow, including in vitro and in vivo treatment groups, dosing schedules, study timeline, and endpoint assessments, is illustrated in Figure 1.

Animal survival was monitored throughout the study period. Animals were monitored for general health status, including body weight, food and water intake, activity, and signs of distress. Animals showing severe deterioration in general condition, persistent inability to eat or drink, markedly reduced activity, or other signs of severe distress were considered to have reached the humane endpoint and were to be euthanized before the scheduled study endpoint. For longitudinal assessments, data obtained before death were retained for analysis, whereas measurements that could not be collected after death were treated as missing and were not replaced. Endpoint analyses included only animals from which endpoint samples were successfully obtained. The onset of hyperglycemia was defined as fasting blood glucose levels of at least 11.1 mmol/L on two successive weekly assessments. Hyperglycemia-free survival was determined from the start of treatment until the first week in which this threshold was fulfilled. Animals that remained below the defined threshold until study completion were censored at the final follow-up time point, whereas animals that died before developing hyperglycemia were censored at their last available fasting blood glucose assessment.

#### 2.3.2. Measurement of Fasting Blood Glucose

For weekly measurement of fasting blood glucose, mice were restrained in a mouse holder, and the tail was disinfected with 70% ethanol. Blood droplets from the tail tips were collected directly onto a glucose test strip. Glucose levels were determined using a glucometer.

#### 2.3.3. OGTT

On the last day before the end of the animal experiment, NOD mice were fasted overnight with access to water. Glucose solution (2 g/kg body weight) was administered by gavage. Blood was collected from the tail to measure glucose levels before gavage (0 min) and at 30, 60, 90, and 120 min following gavage. A glucose time-curve was plotted, with calculation of the AUC using the trapezoidal method to evaluate the effects of drug treatment on glucose tolerance in mice. The area under the oral glucose tolerance test curve was determined using the trapezoidal rule. Blood glucose concentrations obtained at 0, 30, 60, 90, and 120 min were used for AUC estimation according to the following equation: AUC = 30 × [0.5 × G0 + G30 + G60 + G90 + 0.5 × G120] [21], where G0, G30, G60, G90, and G120 denote the glucose values measured at the respective sampling time points. The resulting AUC values, expressed as mmol/L·min, correspond to those generated by trapezoidal integrations in GraphPad Prism.

#### 2.3.4. HE Staining

Pancreatic tissues from NOD mice were fixed in 10% formalin for 48 h, followed by rinsing under running water, dehydration in an ethanol gradient, clearing in xylene, embedding in paraffin, and sectioning (4–7 μm). Sections were kept for 30 min at 65 °C, followed by xylene deparaffinization and rehydration using an ethanol gradient to water. Subsequently, staining of the sections was carried out with hematoxylin and eosin (HE) according to standard procedures, followed by dehydration, xylene clearing, and mounting with neutral resin. Histopathological changes in pancreatic tissues, including islet morphology, structural integrity, and infiltration of inflammatory cells, were evaluated and imaged under light microscopy.

#### 2.3.5. Immunofluorescence Staining

To assess DNA synthesis-associated activity in vivo, BrdU was dissolved in sterile PBS at 10 mg/mL and administered intraperitoneally as a single injection at 50 mg/kg (approximately 100 μL for a 20 g mouse) 24 h before euthanasia. Pancreatic specimens were obtained 24 h after BrdU administration and processed for insulin/BrdU double immunofluorescence analysis. Cells exhibiting BrdU incorporation were considered to have entered the DNA synthesis phase during the labeling interval preceding tissue harvest.

Pancreatic tissue sections underwent deparaffinization, rehydration, and antigen retrieval in citrate buffer (pH 6.0). After blocking with 5% BSA, sections were treated overnight at 4 °C with primary antibodies for dual immunofluorescence detection (insulin in combination with BrdU or cytokeratin-18). Following washing steps, the sections were treated with species-specific fluorophore-conjugated secondary antibodies for 1 h at room temperature with light protection. Nuclei were counterstained with DAPI, and the anti-fade medium-mounted sections were imaged under fluorescence microscopy using identical exposure settings for each channel. Fluorescence-based quantitative analysis was performed using ImageJ/Fiji software. All images were processed under identical parameter settings across experimental groups. Insulin-, BrdU-, and cytokeratin-18-positive signals were quantified from their respective fluorescence channels using uniform channel-specific thresholding criteria. The insulin-positive area was quantified and calculated as the percentage of insulin-positive area relative to the total analyzed field area. One pancreatic section per mouse was included for immunofluorescence evaluation. All distinct insulin-positive islet regions identified within the acquired images were included in the quantitative analysis. Because the number of insulin-positive islet regions varied between sections, no fixed number of islets was selected in advance. Islet regions were included for analysis based on preserved tissue morphology, detectable insulin immunofluorescence signals, and image quality sufficient for threshold-based quantification. For each animal, measurements from individual islet regions were averaged to generate a single value for statistical comparison at the group level. BrdU-positive and cytokeratin-18-positive signals were normalized to the insulin-positive area and expressed as BrdU-positive area/insulin-positive area (%) and cytokeratin-18-positive area/insulin-positive area (%), respectively. Fluorescence image quantification was performed by an investigator blinded to treatment allocation, as described in Section 2.3.8.

#### 2.3.6. TUNEL Assay

Pancreatic tissue sections underwent deparaffinization with xylene, rehydration in an ethanol gradient, and washing with PBS before treatment with proteinase K, followed by washing with PBS. The TUNEL reaction mixture was prepared as directed and applied to the sections, which were then incubated at 37 °C for 60 min with light protection. Counterstaining of the nuclei was carried out with DAPI following washing with PBS. Mounting of the sections was carried out with an anti-fade medium, followed by observation under fluorescence microscopy. TUNEL-positive apoptotic cells were identified by green fluorescence. Fluorescence-based quantitative analysis was performed using ImageJ/Fiji software. TUNEL-positive signal and DAPI-stained nuclei were quantified from their respective fluorescence channels using uniform thresholding criteria and identical analysis settings across all images. The TUNEL-positive area was calculated as the ratio of TUNEL-positive area to DAPI-positive nuclear area and expressed as a percentage (TUNEL/DAPI × 100%). All image analyses were conducted using the same thresholding parameters across all experimental groups. Quantification of TUNEL-positive signals and DAPI-stained nuclear areas was conducted according to the blinded outcome-assessment procedure described in Section 2.3.8.

#### 2.3.7. Histological Assessment of Insulitis

The severity of insulitis was evaluated histologically in paraffin-embedded pancreatic sections. Pancreatic tissues were sectioned at 4–7 μm before staining with H&E, as above. Multiple pancreatic islets from each mouse were examined under a light microscope, and the degree of inflammatory cell infiltration was assessed using a semi-quantitative scoring systems as follows: 0, intact islet without inflammatory cell infiltration; 1, peri-insulitis, with infiltration confined to the islet periphery; 2, infiltration involving less than 50% of the islet area; and 3, severe insulitis, with 50% or more of the islet area infiltrated and marked disruption of islet architecture [22]. At least 10 islets per mouse were evaluated, and the average insulitis score was used for statistical analysis. Islets were included for scoring when their boundaries and inflammatory cell infiltration were assessable. Areas showing tissue folds, tears, inadequate staining, incomplete islet structures, or sectioning artifacts were excluded from analysis. Insulitis scoring was performed using anonymized section codes by an investigator blinded to treatment allocation, as described in Section 2.3.8.

#### 2.3.8. Blinding of Outcome Assessment

To minimize assessment bias, pancreatic tissue sections and microscopy images used for histological and fluorescence-based analyses were anonymized before evaluation. Each section or image was assigned a coded identifier that concealed the corresponding treatment group. The code key linking identifiers to treatment allocations was maintained separately and was inaccessible to investigators responsible for histological scoring and image analysis. H&E-stained sections, TUNEL staining images, and insulin/BrdU or insulin/cytokeratin-18 immunofluorescence images were analyzed using the anonymized identifiers. Investigators performed insulitis scoring, image thresholding, fluorescence quantification, and data recording without knowledge of treatment assignments. Treatment group identities were disclosed only after completion of the predefined scoring and image analysis procedures.

### 2.4. Statistical Analysis

Data are presented as mean ± standard error of the mean (SEM), unless otherwise indicated. Statistical analyses and graphical presentations were performed using GraphPad Prism 10. Data distribution and variance homogeneity were assessed before statistical testing using the Shapiro–Wilk test and Brown–Forsythe test, respectively. When parametric assumptions were satisfied, comparisons among multiple groups were performed using one-way ANOVA followed by Dunnett’s multiple-comparisons test. For datasets with unequal variances, Brown–Forsythe and Welch ANOVA followed by Dunnett’s T3 multiple-comparisons test were applied. Where indicated, additional prespecified Dunnett-adjusted comparisons were performed using each monotherapy group as the reference to compare outcomes with the combination treatment group. For key treatment comparisons, mean differences with 95% confidence intervals (CIs), adjusted *p* values, and descriptive relative changes were reported (Supplementary Table S4); relative changes were calculated as [(treatment group mean − reference group mean)/reference group mean] × 100%. Longitudinal measurements, including body weight and fasting blood glucose, were analyzed using a mixed-effects model with treatment group and time as fixed effects and individual animals as the repeated-measures subject variable. Dunnett’s multiple-comparisons test was used for comparisons between treatment groups and the control group at corresponding time points. Missing values due to animal mortality were not imputed, and all available longitudinal measurements obtained before death were included in the analysis. Endpoint analyses were performed using data from animals with successfully collected corresponding samples. OGTT glucose curves were analyzed using two-way repeated-measures ANOVA, with treatment group as the between-subject factor and time as the within-subject factor, followed by Dunnett’s post hoc comparisons. The area under the curve (AUC) was calculated using the trapezoidal rule and analyzed using one-way ANOVA or Brown–Forsythe and Welch ANOVA, depending on variance homogeneity. MTT assay data were analyzed using two-way ANOVA with treatment and incubation time as factors, followed by Dunnett’s multiple-comparisons test. For endpoints with a 2 × 2 treatment structure, an additional exploratory factorial two-way ANOVA was performed with exendin-4 and rituximab as fixed factors, and the exendin-4 × rituximab interaction term was evaluated. Type III sums of squares were used for unbalanced in vivo datasets. Post hoc treatment-group comparisons within each endpoint were adjusted using the corresponding Dunnett procedure described above. For the exploratory factorial interaction tests across endpoints, the Benjamini–Hochberg procedure was applied to control the false discovery rate (FDR) at 5%. Hyperglycemia-free survival was evaluated using Kaplan–Meier curves, and differences among groups were assessed using the log-rank test. All statistical tests were two-sided. *p* < 0.05 was considered statistically significant, except for the FDR-adjusted interaction analyses, for which q < 0.05 was used.

## 3. Results

### 3.1. Identification and Purity Assessment of Isolated Pancreatic Islets

**Figure 2:**
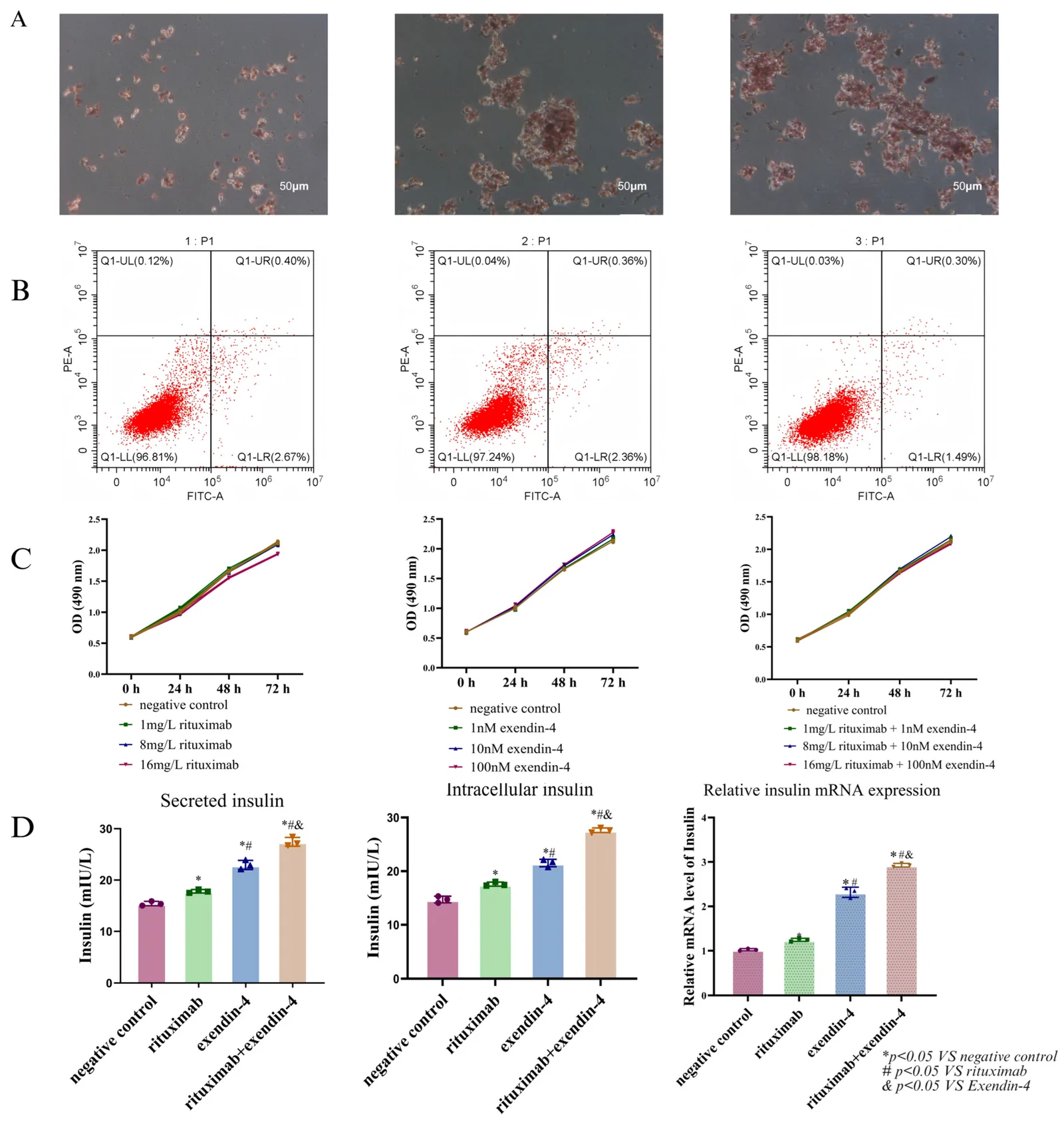
Effect of exendin-4 and rituximab on isolated islet cells in vitro. (**A**) Identification of isolated pancreatic islet cells by dithizone (DTZ) staining. Representative islets showed characteristic red staining. (**B**) Flow cytometric analysis of islet cell viability using Annexin V-FITC/PI staining before treatment. Viable cells are shown in the lower left quadrant, while early apoptotic, late apoptotic, and necrotic cells are indicated in the lower right, upper right, and upper left quadrants, respectively. (**C**) Effects of exendin-4, rituximab, and their combination on metabolic activity, as determined by MTT assay. Exendin-4 at 100 nM produced the highest response among monotherapy groups, whereas 1 mg/L rituximab showed the least reduction in cellular metabolic activity. Among combination treatments, 8 mg/L rituximab plus 10 nM exendin-4 produced the highest MTT signal. (**D**) Effects of exendin-4, rituximab, and their combination on intracellular insulin levels, insulin secretion under high-glucose conditions, and insulin mRNA expression in islet cells. Intracellular insulin and glucose-stimulated secretion were measured by ELISA, and insulin mRNA expression was determined by RT-qPCR. The combination treatment group showed the greatest increase in insulin levels and insulin gene expression.

Primary cells isolated from NOD-scid mice formed clusters of varying sizes. After DTZ staining, most clusters showed distinct red staining under light microscopy, while a small proportion of non-islet cells remained unstained (Figure 2A). These observations indicate successful isolation of pancreatic islet clusters.

### 3.2. Viability of Isolated Pancreatic Islet Cells

Cell viability was assessed using Annexin V-FITC/PI staining and flow cytometry. As shown in Figure 2B, more than 97% of the isolated cells were viable, while apoptotic and necrotic populations each accounted for less than 3%. These results indicate that the isolated islet cells maintained high viability after the isolation procedure and were suitable for downstream experiments.

### 3.3. Effects of Exendin-4 and Rituximab on Cellular Metabolic Activity in Isolated Pancreatic Islets

Metabolic activity following treatment with exendin-4 and rituximab, alone or in combination, was examined using MTT assays. As illustrated in Figure 2C, exendin-4 enhanced MTT signal at all tested concentrations (1, 10, and 100 nM), with the highest increase observed at 100 nM. In contrast, rituximab dose-dependently reduced MTT signal (1, 8, and 16 mg/L), with the least reduction observed at 1 mg/L. In combination treatments, 1 mg/L rituximab plus 1 nM exendin-4 and 8 mg/L rituximab plus 10 nM exendin-4 increased metabolic activity, whereas 16 mg/L rituximab plus 100 nM exendin-4 reduced metabolic activity. Among all combinations, 8 mg/L rituximab plus 10 nM exendin-4 showed the highest metabolic activity (Figure 2C).

### 3.4. Effects of Exendin-4 and Rituximab on Insulin Synthesis and Insulin Gene Expression in Isolated Islet Cultures

After treatment of isolated islet cultures with exendin-4, rituximab, or their combination, intracellular insulin content and insulin secretion under high-glucose conditions (17 mM glucose) were quantified using ELISA. As shown in Figure 2D, higher intracellular and secreted insulin levels were observed in cultures treated with either exendin-4 or rituximab compared with the control cultures, whereas the combined treatment produced the greatest increase. To further assess insulin gene expression, insulin mRNA levels were determined by RT-qPCR. Higher insulin mRNA expression was observed in cultures treated with exendin-4, rituximab, or their combination compared with the control group, with the highest levels found in the combined-treatment group (Figure 2D). Overall, insulin levels and insulin mRNA expression were higher in the treatment groups than in the control group, with the greatest increases observed in the combination group. Given the absence of a CD20-positive B-cell population in the isolated NOD-scid islet system, the in vitro effects associated with rituximab are best interpreted as exploratory findings rather than evidence of a classical B-cell-mediated mechanism of action.

### 3.5. Combination Treatment Lowered Fasting Blood Glucose and Enhanced Oral Glucose Tolerance in NOD Mice

**Figure 3:**
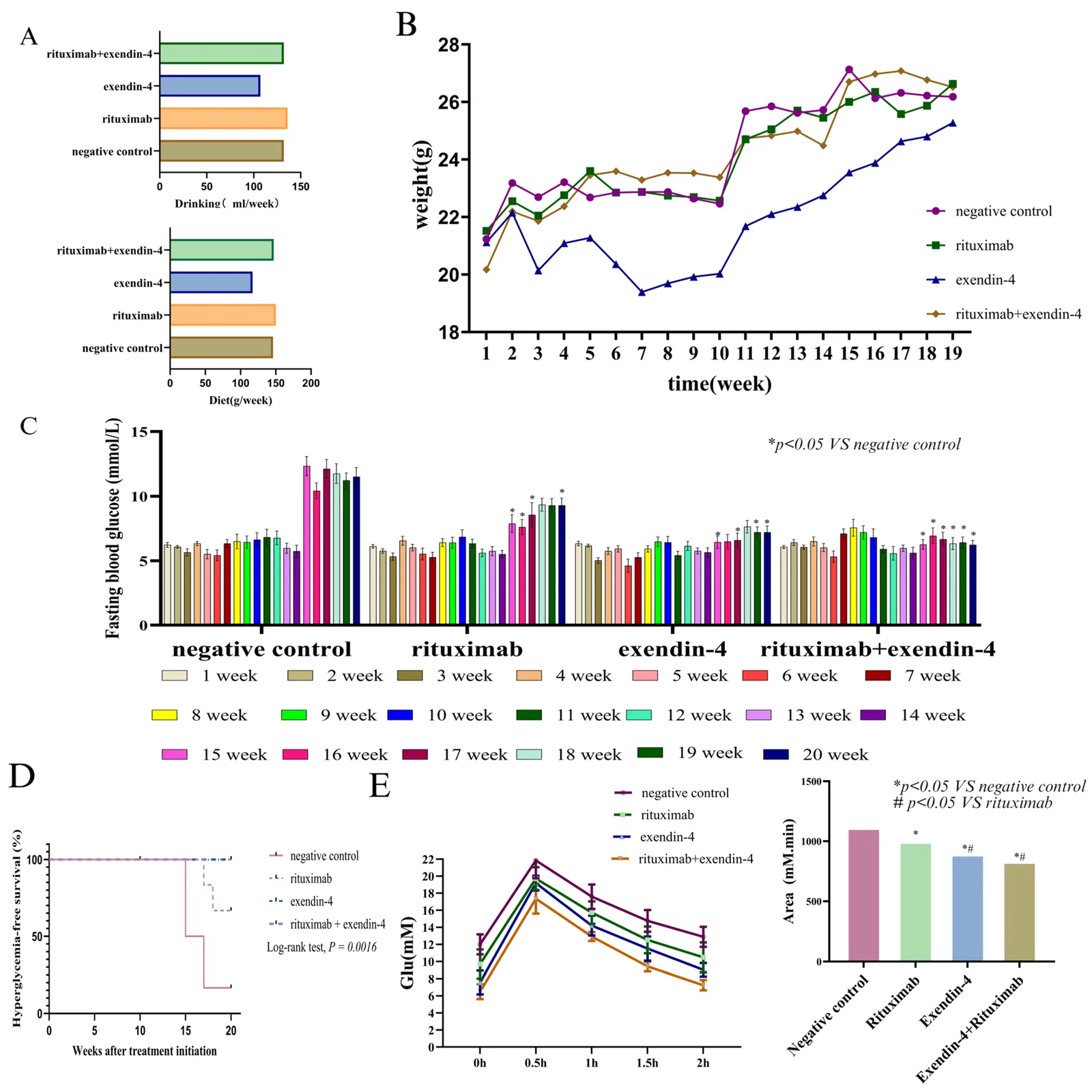
Effects of exendin-4 and rituximab treatment on metabolic parameters in vivo in NOD mice. (**A**) Food and water consumption during the treatment period with no obvious differences among the four groups. (**B**) Body weight changes during the treatment period showing a slight increase over time in all groups, without significant intergroup differences. (**C**) Weekly fasting blood glucose levels during the treatment period, which increased progressively in the control and monotherapy groups, while remaining relatively stable in the combination treatment group. (**D**) Kaplan-Meier analysis of hyperglycemia-free survival over the 20-week treatment period. Hyperglycemia onset was defined as fasting blood glucose ≥ 11.1 mmol/L on two consecutive weekly measurements. Animals that did not reach this endpoint by study completion were censored at the final observation, while those that died before onset were censored at the last recorded fasting blood glucose measurement. Survival curves were compared using the log-rank test (*p* = 0.0016). (**E**) Oral glucose tolerance test (OGTT) and corresponding area under the curve (AUC) after 20 weeks of treatment, showing improved glucose tolerance in the combination treatment group relative to the control and monotherapy groups.

Based on the in vitro findings, the effects of exendin-4 and rituximab were further evaluated in vivo using female NOD mice. Throughout the treatment period, no obvious differences in water and food consumption were observed among the four groups (Figure 3A), and body weight increased slightly over time in all groups without significant intergroup differences (Figure 3B). These results indicate that overall nutritional status and growth were comparable among groups, suggesting that subsequent metabolic changes were not attributable to differences in energy intake or body weight.

At the start of treatment, no animals fulfilled the predefined criterion for hyperglycemia onset, and fasting blood glucose levels did not differ significantly among the four groups. Baseline fasting glucose values for individual mice are presented in Supplementary Table S1. Mice received saline, exendin-4, rituximab, or exendin-4 plus rituximab for approximately 20 weeks. During the treatment period, fasting blood glucose (FBG) in the combination treatment group remained relatively stable without a marked upward trend. In contrast, FBG levels increased progressively in the control, exendin-4, and rituximab groups, and most mice in these groups developed overt hyperglycemia by weeks 15–18 (Figure 3C).

All experimental groups initially consisted of six animals. During the 20-week treatment period, two mice in the exendin-4 group died at week 10, while no mortality was observed in the control, rituximab, or combination treatment groups. The exact causes of these two unexpected deaths could not be determined because no post-mortem diagnostic examination was performed. No overt clinical abnormalities suggestive of treatment-related toxicity were documented before death. Therefore, based on the available observations, a causal relationship between these unexpected deaths and exendin-4 treatment could not be established. Accordingly, the number of animals included in endpoint analyses was 6, 6, 4, and 6 for the control, rituximab, exendin-4, and combination groups, respectively. Longitudinal FBG measurements obtained before death were included in the analysis.

Kaplan–Meier survival analysis based on the predefined hyperglycemia-onset criterion demonstrated significant differences in hyperglycemia-free survival among groups. The combination treatment group showed the highest proportion of hyperglycemia-free animals throughout the study period. Overall group differences were significant according to the log-rank test (Mantel–Cox χ^2^ = 15.34, df = 3, *p* = 0.0016; Figure 3D).

At the end of the study, an OGTT was performed. The glucose AUC in the combination treatment group was markedly lower than that of the control, indicating improved glucose tolerance. Exendin-4 alone and rituximab alone also reduced AUC compared with control, although to a lesser extent than the combination treatment (Figure 3E). Collectively, these findings indicate that combination treatment was associated with delayed hyperglycemia progression and improved oral glucose tolerance in NOD mice.

### 3.6. Combined Treatment Raised Insulin and C-Peptide Levels and Decreased Glucagon Levels in Female NOD Mice

**Figure 4:**
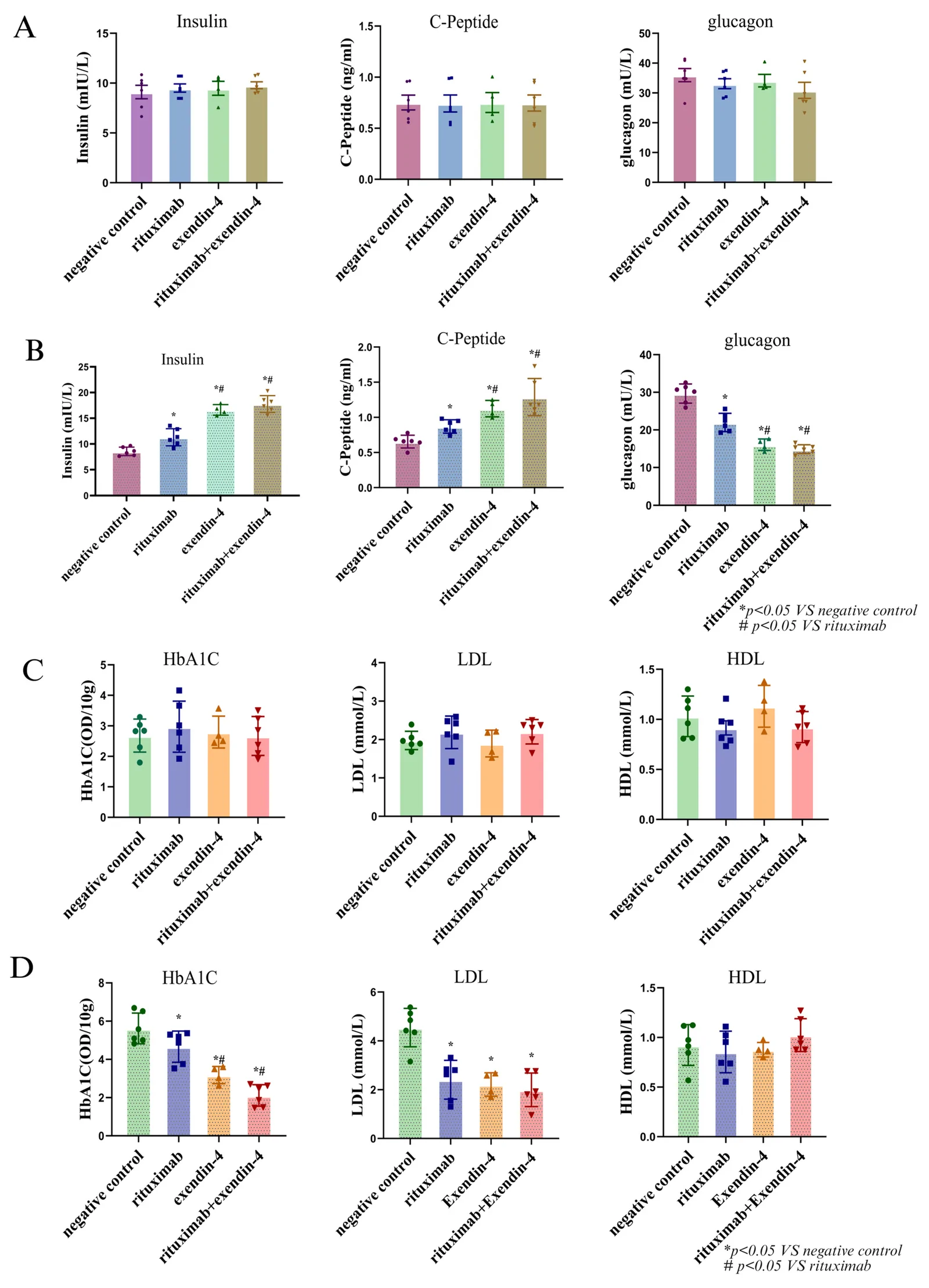
Changes in serum biochemical parameters before and after treatment in NOD mice. (**A**) Baseline serum insulin, C-peptide, and glucagon levels at 9 weeks of age. (**B**) Serum insulin, C-peptide, and glucagon levels after 20 weeks of treatment. (**C**) Baseline glycated hemoglobin (HbA1c), low-density lipoprotein (LDL), and high-density lipoprotein (HDL) levels at 9 weeks of age. (**D**) HbA1c, LDL, and HDL levels after 20 weeks of treatment. Data are presented as mean ± standard error (SE).

C-peptide, a cleavage product generated during insulin biosynthesis, is commonly used as an indicator of endogenous β-cell activity [23]. Serum insulin, C-peptide, and glucagon levels were determined before and after 20 weeks of treatment. No obvious differences in these hormone levels were found among the four groups before treatment (Figure 4A). After 20 weeks, insulin and C-peptide levels were increased in the exendin-4, rituximab, and combination treatment groups relative to the control group, whereas glucagon levels were decreased (Figure 4B). Among the different treatments, the combination treatment showed the most marked effects. Specifically, serum insulin levels reached 11.3, 16.6, and 17.8 mIU/L in the rituximab, exendin-4, and combination treatment groups, respectively. Similarly, C-peptide levels increased to 0.87, 1.12, and 1.29 ng/mL, while glucagon levels decreased to 21.98, 16.06, and 14.88 mU/L, respectively. These results indicate that combination treatment improves circulating islet hormone profiles more effectively than either monotherapy.

### 3.7. Combination Treatment Improves HbA1c and Lipid Profiles in NOD Mice

HbA1c (glycated hemoglobin) reflects long-term glycemic control and is widely used as an indicator of disease progression and therapeutic response in T1DM. In addition, dyslipidemia, particularly elevated low-density lipoprotein (LDL), is commonly associated with T1DM and contributes to metabolic complications of chronic hyperglycemia [24]. Therefore, HbA1c, LDL, and high-density lipoprotein (HDL) levels were evaluated to assess metabolic outcomes following treatment. As shown in Figure 4C, no obvious differences in HbA1c, LDL, or HDL levels were observed among the four groups before treatment. After treatment, HbA1c and LDL levels were reduced in the exendin-4, rituximab, and combination treatment groups relative to the controls, whereas HDL levels did not change significantly (Figure 4D). Among the treatments, the combination treatment resulted in the most marked reductions in HbA1c and LDL levels. These results suggest that combination treatment improved long-term glycemic control and reduced LDL levels in NOD mice.

### 3.8. Histopathological Evaluation of Pancreatic Tissues in NOD Mice

**Figure 5:**
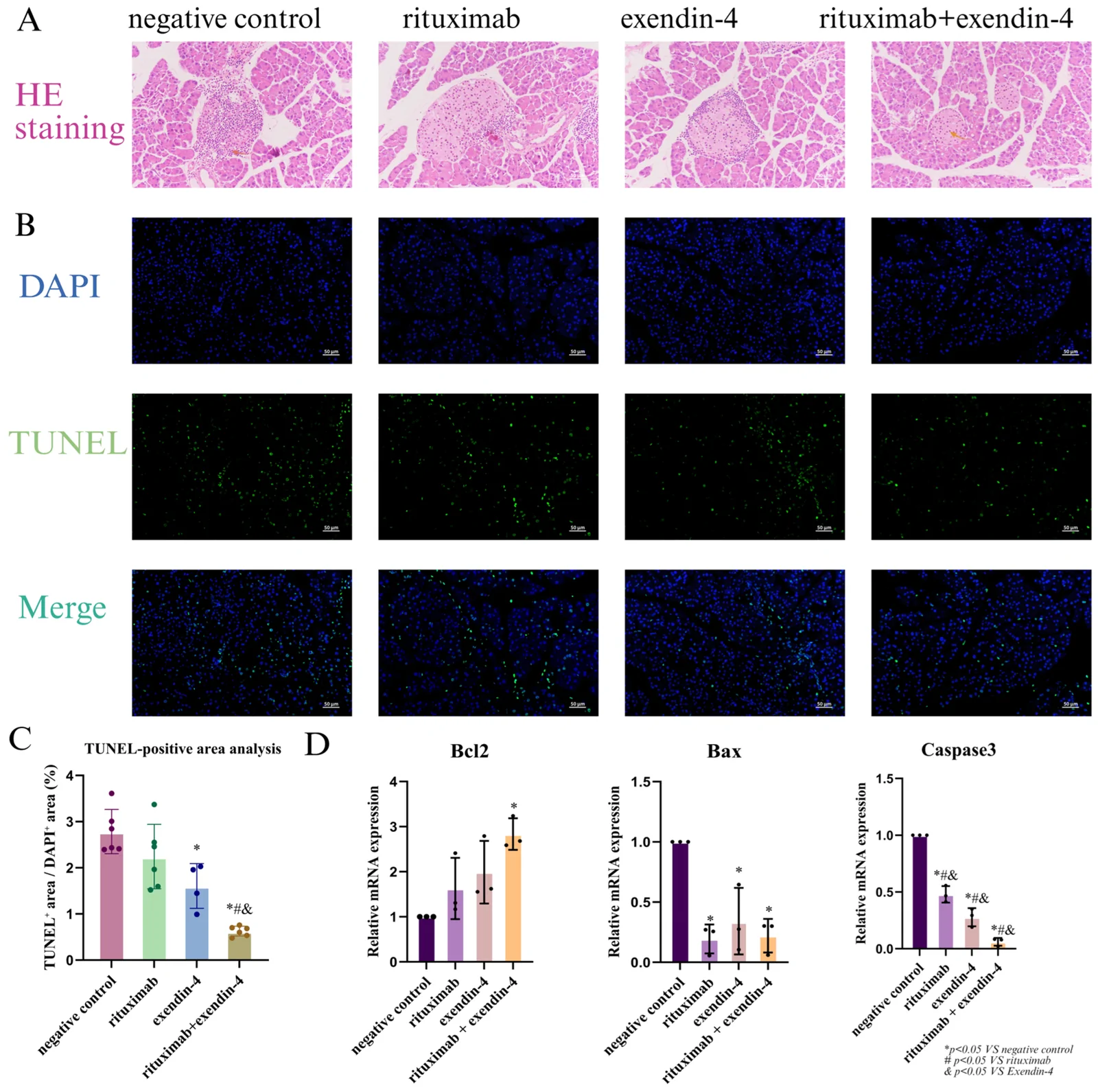
Histopathological changes, apoptosis, and apoptosis-related gene expression in pancreatic tissues of NOD mice after treatment. (**A**) Representative H&E-stained images of pancreatic sections (×200). Red arrows indicate inflammatory cell infiltration, predominantly lymphocytes; yellow arrows indicate pancreatic islets. (**B**) Representative TUNEL staining images of pancreatic sections (×200). TUNEL-positive apoptotic cells are shown by green fluorescence. (**C**) Quantitative analysis of TUNEL-positive area relative to DAPI-stained nuclei area, expressed as TUNEL^+^ area/DAPI^+^ area (%). (**D**) RT-qPCR measurement of apoptosis-related gene levels in pancreatic tissues, including *Bcl-2*, *Bax*, and *Caspase-3*. Compared with the negative control group, treatment groups exhibited raised *Bcl-2* and decreased *Bax* and *Caspase-3* expression to varying degrees. Data are presented as mean ± standard error of the mean (SEM). Statistical significance is indicated as * *p* < 0.05 compared with the negative control; # *p* < 0.05 compared with rituximab, and & *p* < 0.05 compared with exendin-4.

Pancreatic tissues collected after 20 weeks of treatment were sectioned and stained with H&E. As illustrated in Figure 5A, the control group showed substantial inflammatory cell infiltration along with notable disruption of islet architecture. In contrast, the exendin-4 and rituximab monotherapy groups showed comparatively reduced inflammatory infiltration and partial preservation of islet structure. The combination treatment group displayed the mildest histopathological alterations, characterized by minimal inflammatory cell infiltration and well-preserved islet morphology. Semi-quantitative insulitis scoring corroborated these histological findings, demonstrating reduced inflammatory infiltration and improved preservation of islet architecture in the combination treatment group compared with the control and monotherapy groups. Based on the insulitis scoring system, the control group predominantly exhibited grade 2–3 lesions, whereas the exendin-4 and rituximab groups mainly showed grade 1–2 lesions. In contrast, the combination treatment group was predominantly classified as grade 0–1. Collectively, these findings indicate that combination treatment attenuates pancreatic histopathological damage in NOD mice.

### 3.9. Combined Treatment Reduced TUNEL-Positive Staining in Pancreatic Tissues

TUNEL staining was performed on pancreatic sections from NOD mice to evaluate apoptotic changes after treatment. As shown in Figure 5B, the control group exhibited abundant TUNEL-positive cells, indicating a high level of apoptosis in pancreatic tissues. In contrast, both the exendin-4 and rituximab monotherapy groups showed fewer TUNEL-positive cells, suggesting partial reduction of apoptosis. The combination treatment group displayed the weakest TUNEL fluorescence signal and the lowest number of TUNEL-positive cells among all groups. Quantitative analysis in Figure 5C further demonstrated a decrease in the proportion of TUNEL-positive area in the treatment groups, with the most pronounced reduction observed in the combination group. These results indicate that combination treatment was associated with reduced apoptosis-related staining in pancreatic tissues compared with either monotherapy.

### 3.10. Combined Treatment Modulates Apoptosis-Related Gene Expression in Pancreatic Tissues

To further assess apoptosis-associated molecular changes, the mRNA levels of *Bcl-2*, *Bax*, and *Caspase-3* were analyzed by RT-qPCR (Figure 5D). Compared with the control group, exendin-4 or rituximab alone upregulated *Bcl-2* and downregulated *Bax* and *Caspase-3* to varying degrees. The combination treatment group showed the highest levels of *Bcl-2* and the lowest levels of *Caspase-3* among all groups, while *Bax* was also reduced compared with the controls. These changes in expression were consistent with the reduced number of TUNEL-positive cells observed in pancreatic tissues. In conjunction with the decreased number of TUNEL-positive cells, these apoptosis-related transcriptional changes indicate that combination treatment was associated with a shift toward reduced apoptosis activity in pancreatic tissues.

### 3.11. Combined Treatment Increases BrdU- and Cytokeratin-18-Associated Signals in Pancreatic Islets In Vivo

**Figure 6:**
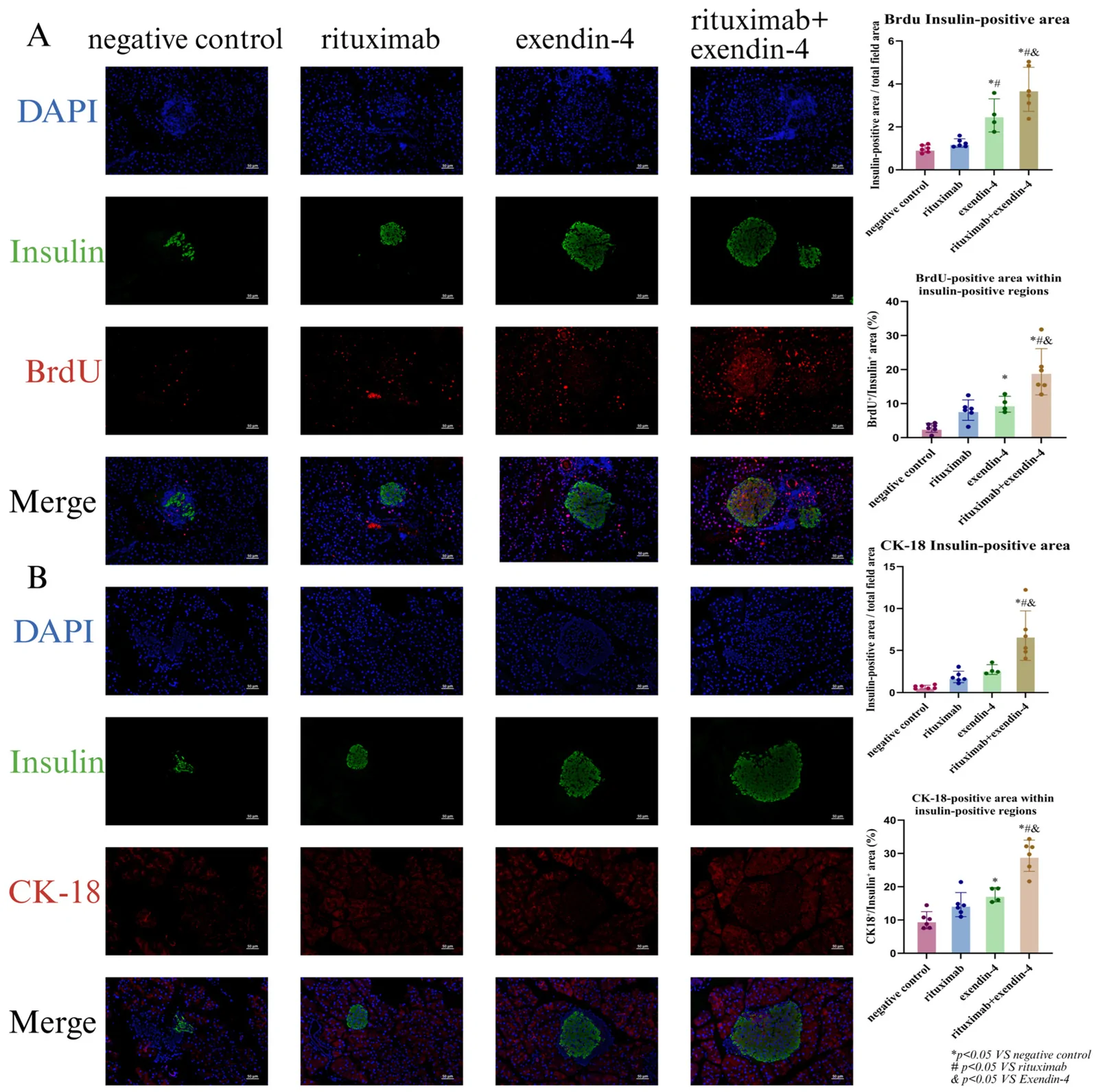
Double immunofluorescence analysis of proliferation- and marker-associated staining in pancreatic tissues after 20 weeks of treatment. (**A**) Representative images of insulin/BrdU double immunofluorescence staining in pancreatic sections from NOD mice, accompanied by quantification of the BrdU-positive area normalized to the insulin-positive area. Insulin-positive cells are indicated in green, whereas BrdU-positive cells are shown in red, and nuclei were counterstained by DAPI staining. Quantitative analyses included the insulin-positive area relative to the total analyzed field area (%) and the BrdU-positive area relative to the insulin-positive area (%). (**B**) Representative images of insulin/cytokeratin-18 double immunofluorescence staining in pancreatic sections from NOD mice, accompanied by quantification of the cytokeratin-18-positive area normalized to the insulin-positive area. Insulin-positive cells are shown in green, cytokeratin-18-positive signals are shown in red, and nuclei were counterstained with DAPI. Quantitative analyses included the insulin-positive area relative to the total analyzed field area (%) and the cytokeratin-18-positive area relative to the insulin-positive area (%). Original magnification, ×200. Data are presented as mean ± SEM. * *p* < 0.05 versus the control group; # *p* < 0.05 versus the rituximab group; & *p* < 0.05 versus the exendin-4 group.

Double immunofluorescence staining was performed on pancreatic sections from female NOD mice after 20 weeks of treatment using anti-BrdU/anti-insulin and anti-cytokeratin-18/anti-insulin antibody combinations (Figure 6). Insulin/BrdU staining was used to assess BrdU incorporation within insulin-positive islet regions, whereas insulin/cytokeratin-18 staining was used to evaluate cytokeratin-18-positive ductal/epithelial marker expression within these regions. Compared with the control group, all treatments resulted in increased insulin fluorescence, with the strongest signals found in the combination treatment group. Quantitative analysis confirmed an increased insulin-positive area relative to the total analyzed field area in the treatment groups, with the greatest increase observed in the combination treatment group, reflecting a greater relative insulin-positive area in the analyzed pancreatic sections. The rituximab monotherapy group also showed increased insulin staining relative to the control group. Moreover, BrdU- and cytokeratin-18-associated fluorescence signals were higher in the treated groups, particularly following the combined treatment. Quantitative analysis confirmed increases in the ratios of BrdU-positive to insulin-positive area and cytokeratin-18-positive to insulin-positive area in the treated groups, with the highest values observed following combination treatment. Merged images further demonstrated more extensive co-localization of staining signals in the combination group compared with other experimental groups. Together with the increased insulin-positive area, the elevated BrdU and cytokeratin-18 signals indicate treatment-associated cellular and epithelial changes within insulin-positive islet regions, accompanying the improved islet phenotype observed following combination treatment.

### 3.12. Additional Analyses of Treatment Effects and Interactions

To further assess potential treatment interactions between exendin-4 and rituximab, an exploratory factorial two-way ANOVA was performed with exendin-4 and rituximab as fixed factors. After Benjamini–Hochberg false discovery rate (FDR) adjustment across endpoints, the exendin-4 × rituximab interaction remained significant for all three in vitro insulin-related endpoints, including secreted insulin (*p* = 0.0266, q = 0.0466), intracellular insulin (*p* = 0.0008, q = 0.0056), and *insulin* mRNA expression (*p* = 0.0008, q = 0.0056). In vivo, significant interactions were observed for serum insulin, HbA1c, TUNEL staining, *Bax* and *Caspase-3* mRNA expression, CK18-positive area/insulin-positive area, and insulitis score. In contrast, the interaction for BrdU-positive area/insulin-positive area did not remain significant after FDR adjustment (*p* = 0.0478, q = 0.0608). No significant interactions were observed for OGTT AUC, C-peptide, or *Bcl-2* mRNA expression (Supplementary Table S3).

Mean differences with corresponding 95% confidence intervals (CIs), adjusted *p* values, and relative changes for the main treatment comparisons are summarized in Supplementary Table S4. These estimates provide additional information on the magnitude of treatment-related differences across metabolic, endocrine, and histopathological outcomes.

## 4. Discussion

Type 1 diabetes mellitus is a consequence of immune-mediated loss of pancreatic β-cells, which leads to insufficient insulin production and sustained hyperglycemia. Because both autoimmune injury and progressive β-cell loss contribute to disease progression, therapeutic strategies that simultaneously modulate immune responses and support residual β-cell function may offer advantages over monotherapy. This study investigated the effects of combination treatment in experimental T1DM using both cellular islet assays and NOD model mice. The findings demonstrated that the combination regimen was more effective than either single-agent treatment in improving insulin secretion, preserving glucose homeostasis, reducing pancreatic injury, suppressing apoptosis, and enhancing islet cell proliferation and marker-associated changes. Exploratory factorial analyses further revealed significant exendin-4 × rituximab interaction terms across several functional and histological outcomes, indicating an endpoint-dependent treatment interaction pattern. The corresponding effect estimates and relative changes also reflected substantial treatment-related differences across several metabolic, endocrine, and histopathological outcomes.

The distinct pharmacological actions of these two agents provide the basis for their combined use. Rituximab, an anti-CD20 monoclonal antibody, targets B-cell-mediated immune injury, which plays a significant role in the autoimmune processes underlying T1DM pathogenesis [20,25,26,27]. In addition to autoantibody production, B cells contribute to antigen presentation and the amplification of islet-directed immune responses. These established pharmacological properties provide the rationale for the use of rituximab in T1DM [28,29]. In contrast, exendin-4, a GLP-1 receptor agonist, primarily acts on β-cell function by enhancing insulin secretion, improving cellular performance, and promoting cell survival. The increase in insulin secretion under high-glucose conditions and the upregulation of *insulin* mRNA following exendin-4-containing treatment are consistent with the known β-cell-supportive effects of GLP-1 receptor agonists. This combined approach integrates the B-cell-directed pharmacological rationale of rituximab with the β-cell-supportive effects of exendin-4. Together, these effects may contribute to the observed reductions in insulitis, improved islet morphology, increased insulin and C-peptide levels, and delayed progression of hyperglycemia following combination treatment in this study. Evidence from previous studies further supports the concept of combining GLP-1 receptor agonists with immune-modulating therapies in T1DM. In diabetic NOD mice, exendin-4 has been shown to improve the efficacy of anti-CD3 monoclonal antibody therapy, leading to improved diabetes remission, glucose tolerance, and insulin responses. Similarly, in individuals with recent-onset T1DM, combined treatment with an anti-IL-21 antibody and liraglutide resulted in superior preservation of stimulated C-peptide compared with placebo, whereas either agent alone showed limited efficacy [13,14]. Consistent with these observations, the present study supports the potential benefit of combining rituximab treatment with β-cell-supportive intervention in an early-stage NOD mouse model, suggesting that this approach may improve metabolic outcomes and is associated with reduced pancreatic inflammatory infiltration on histological assessment.

In vitro experiments showed that exendin-4 enhanced metabolic activity in a concentration-dependent manner, whereas rituximab exerted a suppressive effect at higher concentrations [30], highlighting the importance of dose optimization in combination settings. In the present study, exendin-4-containing treatment increased insulin content and insulin mRNA expression in isolated islet cultures, consistent with the established β-cell-supportive effects of GLP-1 receptor agonists. The rituximab-associated changes observed in vitro should be interpreted with caution. The NOD-scid-derived islet system provided a stable platform for assessing islet functional readouts; however, these mice lack functional T and B lymphocytes and therefore do not replicate the autoimmune microenvironment characteristic of immunocompetent NOD models. Accordingly, the isolated-islet experiments were used to assess islet-level metabolic and insulin-related responses rather than immunomodulatory effects. This limitation is particularly relevant when interpreting the rituximab-related findings. Rituximab is a CD20-targeting monoclonal antibody that mediates its effects primarily through complement-dependent cytotoxicity, antibody-dependent cellular cytotoxicity, and antibody-dependent cellular phagocytosis in B cells [31]; thus, the observed in vitro responses are unlikely to reflect canonical CD20-dependent mechanisms. It should also be noted that isolated islet preparations are not exclusively composed of β-cells. Although NOD-scid mice lack functional adaptive immune cells, residual innate immune cells, including macrophages and dendritic cells, may remain within islet preparations and could influence β-cell functional readouts [32,33]. In addition, as a chimeric IgG1 antibody, rituximab can interact with murine Fcγ receptors [34]; therefore, Fc receptor-mediated interactions with residual innate immune cells represent a possible indirect explanation for the observed metabolic and insulin-related changes. In contrast, complement-mediated effects are less likely in this system because no CD20-positive target population was confirmed and no exogenous complement was added. Because these possibilities were not directly examined, the rituximab-associated in vitro findings are considered exploratory and should be interpreted as culture-level treatment-associated responses rather than evidence of a direct effect on β-cells.

The in vivo results further support a beneficial effect of the combined regimen. Compared with the control and monotherapy groups, NOD mice treated with exendin-4 plus rituximab exhibited more stable fasting blood glucose levels, improved oral glucose tolerance, increased insulin and C-peptide levels, reduced glucagon levels, and lower HbA1c and LDL levels. These findings indicate improved β-cell secretory function and overall metabolic control. HDL levels remained unchanged following treatment, indicating that the metabolic benefits of the combined regimen were primarily reflected in improved glycemic control and reduced LDL levels rather than a generalized correction of all lipid parameters. This observation aligns with previous reports showing that HDL-C levels in T1DM may be normal or elevated, whereas dyslipidemia-related risk is more strongly associated with LDL abnormalities and qualitative alterations in lipoprotein function [35,36]. Importantly, these functional and metabolic improvements should be distinguished from structural preservation and changes in the relative extent of insulin-positive islet regions, which were assessed separately by histological and immunofluorescence-based analyses. Histological analysis showed better preservation of islet architecture and reduced inflammatory cell infiltration in the combination treatment group compared with the control and single-treatment groups, suggesting attenuation of pancreatic tissue injury. However, the specific immune-cell populations underlying these histological changes were not directly characterized in the present study. Further immune-cell profiling will be required to define the specific immunological contribution of rituximab to these in vivo effects. Collectively, these observations indicate that the combined strategy was more effective than monotherapy in maintaining islet structure and metabolic function in NOD mice.

Another notable observation was a reduction in apoptosis associated with the combination treatment. TUNEL staining showed fewer apoptotic cells in pancreatic tissues, with the combination -treatment group exhibiting the lowest counts. RT-qPCR analysis further demonstrated upregulation of *Bcl-2* alongside downregulation of *Bax* and *Caspase-3*. These changes reflect a shift in the expression pattern of apoptosis-related genes toward a pro-survival state. The consistency between histological and molecular findings supports an association between the combined treatment and reduced islet apoptosis in vivo. However, because these observations were derived from TUNEL staining and mRNA expression analyses, they should be interpreted as indicators of apoptosis-associated changes rather than definitive evidence of modulation at the protein level. Quantitative immunofluorescence analysis also showed a greater insulin-positive area relative to the total analyzed field area in the treatment groups, with the largest increase observed following combination treatment. This measurement reflects the relative extent of insulin-positive islet regions within the analyzed pancreatic sections rather than absolute β-cell mass. Thus, the present findings support improved endocrine function together with better preservation of islet architecture and a greater relative insulin-positive area, although absolute β-cell mass was not directly quantified. In addition, double immunofluorescence staining revealed increased insulin/BrdU and insulin/cytokeratin-18 signals in the combination group. When considered together with the greater relative insulin-positive area, improved insulin secretion, and better metabolic control, these findings suggest that combination treatment was associated with a more favorable islet phenotype involving enhanced proliferation-associated activity and epithelial marker-associated changes. BrdU incorporation has been widely used as a proliferation-associated readout in pancreatic β-cell studies, including studies demonstrating exendin-4-induced β-cell proliferation [37,38,39]. Accordingly, the increased insulin/BrdU signal observed in the present study supports enhanced proliferation-associated activity within insulin-positive islet regions following treatment. Cytokeratin-18 was interpreted as a ductal/epithelial phenotype-associated marker, as cytokeratins are widely used to characterize pancreatic ductal epithelial differentiation and have also been investigated in the context of islet neogenesis [40]. Accordingly, in this study, insulin/cytokeratin-18 staining was used as a ductal/epithelial phenotype-associated readout to complement BrdU-based proliferation assessment. The increased insulin/cytokeratin-18 signal may therefore suggest concurrent ductal/epithelial phenotype-associated alterations accompanying treatment-induced islet responses. Previous studies have proposed pancreatic ductal epithelium as a potential source of β-cell neogenesis, and lineage-tracing evidence has suggested that Ngn3-expressing ductal cells may contribute to adult β-cell formation [41]. However, the extent to which ductal cells contribute to β-cell neogenesis in adulthood remains controversial [42,43]. Therefore, the increased insulin/BrdU and insulin/cytokeratin-18 signals support treatment-associated proliferation-related and ductal/epithelial responses within insulin-positive islet regions. Cytokeratin-18 provides complementary epithelial context for these changes, while additional proliferation and lineage-specific markers, such as Ki67, PDX1, NKX6.1, MAFA, or NeuroD1, together with lineage-tracing approaches, would help further determine whether these responses involve β-cell regenerative processes.

Despite these findings, several limitations should be considered. First, the molecular mechanisms underlying the combined treatment were not fully defined. The endpoint-dependent interaction pattern observed in the factorial analyses further highlights the need to clarify the biological relationship between the two treatments. In particular, key regulators of β-cell function and immune tolerance were not directly examined. Given the central role of immune dysregulation in T1DM, future studies should investigate pathways involved in β-cell maintenance and immune regulation, including PDX-1, which is closely associated with insulin expression and β-cell differentiation [44,45], as well as Foxp3-related pathways that are important for regulatory T-cell function and immune tolerance [46,47]. Future studies integrating comprehensive immune profiling, cytokine characterization, and detailed assessments of β-cell function will be important to further elucidate the mechanisms underlying the metabolic and histopathological effects of combination therapy. Evidence from antigen-specific immunotherapy studies further supports the importance of restoring immune regulation in T1DM [48]. Second, the analysis of apoptosis-related changes was based on TUNEL staining and mRNA expression of *Bcl-2*, *Bax*, and *Caspase-3*; therefore, confirmation at the protein level, including cleaved Caspase-3 and quantification of the Bcl-2/Bax ratio, will be necessary in future studies. Third, the in vivo experiments included six NOD mice per group, and no formal a priori power calculation was performed. The observed metabolic and histological differences provide preliminary evidence of treatment effects, but confirmation in larger cohorts will be important, particularly for outcomes related to hyperglycemia incidence and endpoint measures. Furthermore, no dedicated in vivo dose-escalation study was performed before the main experiment; accordingly, future work should explore multiple dosing regimens to determine the optimal therapeutic window, safety profile, and administration strategy. Fourth, although the present findings support the potential benefit of combining β-cell-supportive intervention with immune modulation in NOD mice, their translation to clinical applications requires careful consideration. Rituximab has been reported to transiently preserve β-cell function in patients with recent-onset T1DM; however, its overall clinical benefit remains limited and does not support its use as a standard long-term therapeutic option [49]. Similarly, GLP-1 receptor agonists used as adjunctive therapy in T1DM have demonstrated metabolic benefits, including reduced insulin requirements and body weight, but have also been associated with safety concerns such as hypoglycemia, gastrointestinal adverse events, and ketosis in certain clinical contexts [50,51]. Therefore, the combined strategy of the combined strategy of B-cell-targeted therapy and GLP-1 receptor agonism should be regarded as a preclinical proof-of-concept rather than a clinically established regimen. Other considerations, including infection risk related to immune modulation, glycemic variability during insulin adjustment, cost-effectiveness, patient adherence, and long-term safety, require systematic evaluation before clinical translation. Future studies should therefore aim to define optimal dosing strategies, safety margins, and responsive patient subgroups most likely to benefit from combined immunomodulatory and β-cell-supportive interventions. GLP-1 receptor agonists are typically administered by injection, which may limit long-term acceptability in the management of chronic metabolic diseases. Therefore, optimization of administration strategies remains an important consideration for future development. In this context, Opt-rolGLP-1, a novel orally available GLP-1 analogue with enhanced resistance to enzymatic degradation and an extended half-life, may represent a more clinically practical alternative for translational application [52,53]. Finally, these results were obtained from NOD mice and isolated islet cultures, and the study did not directly characterize immune-cell populations, including pancreatic B cells and CD4+/CD8+ T-cell infiltration, autoantibody levels, or inflammatory cytokine profiles. Future investigations incorporating immunophenotyping of B cells, T-cell subsets, regulatory T cells, and macrophages, together with cytokine characterization, will be important to further define the immunological mechanisms contributing to the combined-treatment effects. Direct assessment of downstream GLP-1 receptor signaling will also be important to further clarify the contribution of exendin-4 to the combined-treatment response. Assessments, including insulin tolerance testing and dynamic GSIS or islet perifusion assays, may help distinguish alterations in insulin sensitivity from changes in β-cell secretory function and provide further insight into the therapeutic potential of this approach.

## 5. Conclusions

Combined exendin-4 and rituximab treatment demonstrated greater therapeutic efficacy than either monotherapy in experimental T1DM. The observed effects were associated with better preservation of islet architecture and delayed progression of hyperglycemia in NOD mice, providing preliminary preclinical support for further investigation of this combination strategy as a potential early-intervention approach.

## Figures and Tables

**Table 1:** Primer sequences and RT-qPCR performance parameters.

Genes	Strand	Primer Sequences	Amplicon Size (bp)	Amplification Efficiency (%)	R^2^
β-actin	Forward	CGAGGACTTTGATTGCACATTG	83	98.6	0.998
	Reverse	AGAGAAGTGGGGTGGCTTTTAG			
*Insulin*	Forward	CGTGGCTTCTTCTACACACCCA	134	96.7	0.997
	Reverse	TGCAGCACTGATCCACAATGCC			
*Bcl2*	Forward	CCTGTGGATGACTGAGTACCTG	123	101.4	0.999
	Reverse	AGCCAGGAGAAATCAAACAGAGG			
*Bax*	Forward	AGACAGGGGCCTTTTTGCTA	137	95.9	0.998
	Reverse	AATTCGCCGGAGACACTCG			
*Caspase-3*	Forward	GGAGTCTGACTGGAAAGCCGAA	113	99.2	0.996
	Reverse	CTTCTGGCAAGCCATCTCCTCA			

## Data Availability

The data that support the findings of this study are available from the corresponding author upon reasonable request.

## Supplementary Materials

The following supporting information can be downloaded at https://www.mdpi.com/article/10.3390/biology15171472/s1, Table S1: Weekly fasting blood glucose records of individual mice during the 20-week treatment period; Table S2: Exact *p* values for statistical comparisons presented in the main figures; Table S3: Results of exploratory factorial two-way ANOVA and FDR-adjusted interaction analyses; Table S4: Mean differences, 95% confidence intervals, adjusted *p* values, and relative changes for the main treatment comparisons.

## Abbreviations

The following abbreviations are used in this manuscript:

T1DM	Type 1 diabetes mellitus
NOD	Non-obese diabetic
GLP-1	Glucagon-like peptide-1
FBG	Fasting blood glucose
OGTT	Oral glucose tolerance test
AUC	Area under the curve
HbA1c	Glycated hemoglobin
LDL	Low-density lipoprotein
HDL	High-density lipoprotein
BrdU	5-Bromo-2′-deoxyuridine
CK18	Cytokeratin-18
TUNEL	Terminal deoxynucleotidyl transferase dUTP nick end labeling
RT-qPCR	Reverse transcription quantitative polymerase chain reaction
FDR	False discovery rate
SEM	Standard error of the mean
